# Costs of Three *Wolbachia* Infections on the Survival of *Aedes aegypti* Larvae under Starvation Conditions

**DOI:** 10.1371/journal.pntd.0004320

**Published:** 2016-01-08

**Authors:** Perran A. Ross, Nancy M. Endersby, Ary A. Hoffmann

**Affiliations:** Bio21 Institute and the School of BioSciences, University of Melbourne, Parkville, Victoria, Australia; Mahidol University, THAILAND

## Abstract

The mosquito *Aedes aegypti*, the principal vector of dengue virus, has recently been infected experimentally with *Wolbachia*: intracellular bacteria that possess potential as dengue biological control agents. *Wolbachia* depend on their hosts for nutrients they are unable to synthesize themselves. Consequently, competition between *Wolbachia* and their host for resources could reduce host fitness under the competitive conditions commonly experienced by larvae of *Ae*. *aegypti* in the field, hampering the invasion of *Wolbachia* into natural mosquito populations. We assess the survival and development of *Ae*. *aegypti* larvae under starvation conditions when infected with each of three experimentally-generated *Wolbachia* strains: *w*Mel, *w*MelPop and *w*AlbB, and compare their fitness to wild-type uninfected larvae. We find that all three *Wolbachia* infections reduce the survival of larvae relative to those that are uninfected, and the severity of the effect is concordant with previously characterized fitness costs to other life stages. We also investigate the ability of larvae to recover from extended food deprivation and find no effect of *Wolbachia* on this trait. *Aedes aegypti* larvae of all infection types were able to resume their development after one month of no food, pupate rapidly, emerge at a large size, and exhibit complete cytoplasmic incompatibility and maternal transmission. A lowered ability of *Wolbachia*-infected larvae to survive under starvation conditions will increase the threshold infection frequency required for *Wolbachia* to establish in highly competitive natural *Ae*. *aegypti* populations and will also reduce the speed of invasion. This study also provides insights into survival strategies of larvae when developing in stressful environments.

## Author Summary

Dengue is currently the most important arboviral disease in the world. With no effective treatment or commercial vaccine available, strategies to control dengue focus on its mosquito vectors, primarily *Aedes aegypti*. A recent effort to reduce the burden of dengue aims to replace native *Ae*. *aegypti* with those refractory to the virus. This is achieved by infecting mosquitoes with *Wolbachia*, bacteria which can invade insect populations by exploiting host reproduction. Some strains of *Wolbachia* have harmful effects on the mosquito host which can inhibit its ability to spread. While these costs have been characterized comprehensively in the laboratory, we must also consider any impacts when mosquitoes experience stresses that commonly occur in nature. For instance, *Ae*. *aegypti* larvae often develop in highly-occupied habitats where food is scarce. We investigated the effects of *Wolbachia* on mosquito larvae when they develop under extremely nutrient-limited conditions and found costs to survival for all strains. This will translate to a reduced ability of *Wolbachia*-infected mosquitoes to replace native populations in competitive habitats.

## Introduction

Dengue fever is an increasing threat to global health. An estimated 50 to 390 million new cases of dengue occur annually, with 2.5 billion people living in areas at risk of infection [1,2]. At present, dengue lacks an effective treatment or vaccine that protects against all serotypes of the virus. Thus, strategies to reduce infection incidence must rely on the control of its mosquito vector, principally *Aedes aegypti* [3,4]. While permanent eradication is unlikely to be achieved, many emerging genetic and biological approaches aim to reduce mosquito vectorial capacity [5,6].

A promising new approach to dengue control utilizes the obligate intracellular bacterium, *Wolbachia*. *Wolbachia* are maternally inherited [7] and usually manipulate the reproduction of their hosts to enhance their own transmission [8]. The most common manipulation induced by *Wolbachia* is cytoplasmic incompatibility; a mechanism where embryonic lethality occurs when an infected male mates with a female that is not infected with *Wolbachia*, providing infected females with a relative reproductive advantage [9,10]. Many *Wolbachia* infections also provide protection to their host against pathogens, including RNA viruses [11–13]. These traits have enabled *Wolbachia* to be implemented in strategies to both suppress [14,15] and replace [16–18] insect populations.

While *Ae*. *aegypti* does not harbour a natural *Wolbachia* infection [19,20], three infections have been stably introduced into the vector: the *w*MelPop and *w*Mel strains originating from *Drosophila melanogaster* [21,22] and *w*AlbB from the mosquito *Aedes albopictus* [23]. All three infections are transmitted vertically at high rates and exhibit complete cytoplasmic incompatibility [21–23], and these effects have remained stable after many years in the novel host [24–26]. Crucially, they also suppress the replication and transmission of dengue virus in *Ae*. *aegypti* [22,27,28], giving them potential to reduce dengue incidence in transformed populations. Establishment of *Wolbachia* in a field population is facilitated largely by maternal transmission and cytoplasmic incompatibility [16,29,30]. However, because *Wolbachia*-infected mosquitoes must survive and reproduce in competition with the native inhabitants, lower relative fitness of infected mosquitoes can hamper the invasibility of *Wolbachia* [31–33].

The experimental *Wolbachia* infections established in *Ae*. *aegypti* vary considerably in their effects on mosquito life-history traits. The *w*Mel infection is relatively benign and has invaded both caged [22] and field [18] populations. *w*Mel remains at a high frequency in mosquitoes collected from the release sites, three years after releases of *w*Mel ceased in two suburbs of Cairns, Australia[24]. Conversely, the *w*MelPop infection tends to overreplicate in host cells, leading to rapid tissue degeneration and early death [34–36]. It exacts a high fitness cost on *Ae*. *aegypti*; *w*MelPop shortens adult lifespan [21,37], while fecundity [38], blood feeding success [39,40] and quiescent egg viability [37,38,41] deteriorate rapidly with age. *w*MelPop also modifies behaviour and metabolism [42], reduces the response of larvae to light stimulation [43], delays larval development, and decreases viability and adult size when reared under crowded conditions [44]. The *w*AlbB infection has intermediate fitness costs, likely due to its moderate density in host tissues that lies between that of *w*Mel and *w*MelPop [26].

While each of these infections can invade caged populations of *Ae*. *aegypti* [22,23,26], the mosquitoes were not exposed to many of the selective pressures that exist in the field [6]. Suitable habitats for immature development in the field are limited; as a consequence, larvae are often subjected to competition for space and nutrition [45–48]. Though *Wolbachia* infection has no clear effect on *Ae*. *aegypti* larval development in the absence of stress [22,26,37,38], some costs emerge when larvae are crowded [44]. Many fitness costs of *Wolbachia* in *Ae*. *aegypti* also tend to become clearer with age in both adults and eggs [26,37,39]. As larval development times can reach several weeks, or even months in the field [49] and often experience periods of food limitation [47,48], deleterious effects of *Wolbachia* on larvae undetected in laboratory studies could emerge when development times are prolonged, impacting *Wolbachia*’s invasive potential. This could explain a lack of invasion success by *w*MelPop in natural populations despite multiple attempts to establish the infection in the field [50].

*Aedes aegypti* larvae are adapted to nutrient poor-habitats as food limitation is a major regulator of their population size [47,51]. Larvae decrease their rate of development in response to food scarcity, delaying metamorphosis until reaching a critical threshold of nutritional reserves [52–55], and larvae can resist starvation for several weeks at a time [51,56–58]. This is achieved largely by expending their accumulated reserves [59–61], though larvae also scavenge on dead conspecifics [62,63] and may even prey on younger larvae [64] to increase their chance of survival. *Wolbachia* depend on their hosts for a wide range of resources they cannot synthesize themselves [65–68]. Since *Wolbachia* increase the activity and metabolic rate of *Ae*. *aegypti* in adults, at least for the *w*MelPop infection [42], we hypothesize that *Wolbachia* may also increase the rate at which energy reserves are depleted in larvae without food. *Aedes aegypti* breeding containers typically have low productivity and high food intermittency because leaf litter, animal detritus and the microorganisms that break them down are the primary source of nutrition [62,69,70]. Thus, the ability to survive periods of limited food is a critical aspect of larval fitness [47,51]. In the field, competition between *Wolbachia* and *Ae*. *aegypti* for resources could substantially reduce the survival of larvae, limiting the potential for *Wolbachia* to invade and persist in natural populations.

In this study we investigate the effects of *w*Mel, *w*AlbB and *w*MelPop infection on the ability of *Ae*. *aegypti* larvae to survive and develop under extreme nutrient stress. We compare the survival and development of *Wolbachia*-infected and uninfected larvae under starvation conditions when held in groups, when infected and uninfected larvae are together in the same container, or when isolated, and test their ability to recover when an influx of resources is provided. We also examine the ability of *Wolbachia* to express their reproductive effects when *Ae*. *aegypti* larvae are held under starvation conditions for extended periods. We then consider the likely impact of any fitness costs imposed by *Wolbachia* on the potential for these infections to invade highly competitive populations.

## Methods

### Colony maintenance and mosquito strains

*Aedes aegypti* mosquitoes were sourced from Cairns, Queensland and maintained under laboratory conditions for at least two generations before use in experiments. *Wolbachia*-infected lines were generated by crossing male uninfected Cairns mosquitoes to laboratory-reared female mosquitoes infected with *w*Mel [22], *w*AlbB [23] or *w*MelPop [21] to maintain a similar genetic background (>98%) between colonies. Mosquitoes were kept in the laboratory at 26°C ± 1°C and 80–90% relative humidity with a 12:12 light: dark photoperiod, and maintained according to methods described by Axford et al. [26]. Within one week of emerging, female adults were allowed to feed to repletion on the forearm of a single human volunteer. Blood feeding of female mosquitoes on human volunteers for this research has been approved by the University of Melbourne Human Ethics Committee (approval 0723847). All adult subjects provided informed written consent (no children were involved).

### Rearing regime

Larvae were reared under a common regime before initiating the food-deprivation period for all experiments. At the beginning of each experiment, *w*Mel-infected, *w*MelPop-infected, *w*AlbB-infected and uninfected eggs were hatched synchronously in separate trays containing 3 L of RO (reverse osmosis) water, 2–3 grains of yeast and one crushed tablet of TetraMin tropical fish food (Tetra, Melle, Germany). Within three hours of hatching, cohorts of 200 1^st^ instar larvae were transferred to plastic trays filled with 700 mL of RO water and fed TetraMin *ad libitum* for 72 hours. This rearing environment was chosen as development times do not differ significantly between *Wolbachia*-infected and uninfected larvae with abundant nutrition at this density. After the feeding period, larvae were pipetted into fresh trays of RO water. To remove any remaining food particles, larvae were rinsed by passing them through two additional trays of water before being added to experimental containers. All experiments used 72 hour old 3^rd^ instar larvae of approximately the same size, and were conducted at 26°C ± 1°C and 80–90% relative humidity with a 12:12 light: dark photoperiod.

### Survival of isolated larvae under starvation conditions

We tested the ability of *Wolbachia*-infected and uninfected larvae to survive starvation conditions in the absence of conspecific larvae, removing any effects of resource competition and also the ability to scavenge on dead larvae. Two independent experiments were conducted; in each, 96 larvae per infection type (see *rearing regime*) were added individually to wells of Costar 12-well cell culture plates (Corning, Corning, NY) filled with ~4 mL of RO water only. Plates were enclosed in stockings and held in a tray covered with a mesh lid to minimize external sources of food input, and RO water was topped up daily to counter evaporation. For both experiments, wells were monitored for mortality daily until all larvae had died. A larva was considered dead when no movement was observed after fifteen seconds of physical stimulation.

In the first experiment, plates were unmanipulated with the exception of maintaining a consistent volume of water in each well. In the second experiment, water was replaced completely twice per week to reduce the accumulation of microorganisms as a potential source of nutrition (e.g., bacteria, algae, protozoa, fungi) and waste in the water [73]. For this experiment, larvae were removed from wells and rinsed by pipetting through multiple trays of RO water, then returned to wells filled with a fresh change of water.

### Survival and development of larvae held in groups under starvation conditions

Two independent experiments tested the ability of *Wolbachia*-infected and uninfected larvae to survive starvation conditions when held in the presence of conspecific larvae. Larvae (see *rearing regime*) were added to circular plastic containers (9.5–11.5 cm radius, 7 cm height) with mesh lids and filled with 200 mL of RO water only (no TetraMin was provided). Mortality was scored every second day by temporarily pipetting larvae into a separate container of RO water. Numbers of dead and live larvae were counted before all larvae (including dead larvae) were returned to the original container. Water was refreshed every four days by transferring all larvae to a new container of RO water. In the first experiment, larvae were added to containers in groups of 50. Each container was replicated eight times for the uninfected, *w*Mel, *w*AlbB and *w*MelPop strains. The experiment was terminated when all larvae had died or had reached adulthood.

During field releases, preferential mortality of *Wolbachia*-infected larvae in nutrient-deprived containers could release the remaining larvae from food stress, providing an advantage to uninfected larvae [71,72]. A second experiment was therefore conducted to determine whether there were differences in survival when *Wolbachia*-infected and uninfected larvae were held together in mixed proportions within the same container. Cohorts of larvae were added to plastic containers filled with 200 mL of RO water in the following proportions (*Wolbachia*-infected to uninfected): 12:36, 24:24 and 36:12. Additional cohorts of 48 *Wolbachia*-infected and 48 uninfected larvae were set up as controls. Treatments (mixed proportions) were replicated eight times each, while the controls (pure cohorts) were replicated four times, and the experiment was repeated for the *w*Mel, *w*AlbB and *w*MelPop infections. Containers were monitored as per the previous experiment, with the exception that the five longest surviving larvae in each container were removed and screened for their *Wolbachia* infection status (see *DNA extraction and Wolbachia detection*). The proportion of individuals infected with *Wolbachia* in the longest surviving larvae was then compared with the initial proportion of larvae infected with *Wolbachia* in each container (see *statistical analysis*).

In both experiments, a few percent of larvae were able to reach the pupal and adult stages due to the availability of dead conspecific larvae as a food resource. All adults emerging throughout the two group experiments were stored in ethanol for wing length measurement and later tested for their *Wolbachia* infection status (see *wing length measurements* and *DNA extraction and Wolbachia detection*). Their development time and sex were also recorded.

### Recovery from food deprivation

An experiment was carried out to test the ability of *Wolbachia*-infected and uninfected larvae to recover from starvation conditions after providing an influx of resources. Larvae (see *rearing regime*) were added to RO water in groups of 50 (see *survival and development of larvae held in groups under starvation conditions*). Containers were then divided into two treatments; larvae were re-fed TetraMin *ad libitum* after either 15 or 25 days of surviving starvation conditions. These two time points were chosen based on when substantial starvation-induced mortality had occurred; approximately 25% and 10% of larvae were remaining on Days 15 and 25 respectively (S1 Fig). For each infection type and treatment, the following observations were recorded: the number of surviving larvae upon the resumption of feeding, rates of pupation and survival to the pupal stage after re-feeding, rates of adult emergence and survival to adulthood, and the body size (see *wing length measurements*) and sex ratio of emerging adults. Containers were replicated between six and eight times for each infection type and treatment.

### Cytoplasmic incompatibility when larvae are food-deprived then re-fed

We ran a series of experiments to determine if the reproductive effects caused by *Wolbachia* remain robust when larvae are held under starvation conditions for an extended period. To test the level of cytoplasmic incompatibility induced by *Wolbachia*-infected males, larvae (see *rearing regime*) were added to containers of RO water and their development was suspended for ~30 days by maintaining them in the absence of TetraMin. After this period larvae were again fed TetraMin *ad libitum* until pupation. Pupae were sexed (males are smaller than females), and male pupae pipetted into small cups of RO water and allowed to emerge in 1.5 L plastic containers with mesh sides and a stocking lid. Female pupae emerging from this treatment were set aside for an additional experiment on reproductive effects (see *fecundity and maternal transmission*). After confirming the sex of all males as adults, newly-emerged uninfected females that were reared under standard laboratory conditions (see *colony maintenance and mosquito strains*) were added to each cage and allowed to mate freely with *Wolbachia*-infected males. Seven *Wolbachia*-infected males and seven uninfected females were held in each experimental cage, and crosses were replicated eight times for the *w*Mel, *w*AlbB and *w*MelPop infections. Cages of adults were provided access to 10% sucrose solution and water throughout the experiment. Crosses between standard laboratory-reared *Wolbachia*-infected males and uninfected females were set up as controls, as these crosses are known to produce no viable offspring [21–23]. Females were then blood fed and eggs were collected according to Axford et al. [26] for three gonotrophic cycles.

### Maternal transmission and fecundity when larvae are food-deprived then re-fed

This experiment assessed the rate at which *Wolbachia*-infected females transmit the infection to their offspring when their development time is greatly extended. Food-deprived and re-fed larvae from the *w*Mel, *w*AlbB and *w*MelPop lines (see *cytoplasmic incompatibility*) were sorted by sex, and 100 females per infection type were added to 12 L plastic cages and provided with 10% sucrose solution and a source of water. 100 uninfected males reared under standard laboratory conditions were then aspirated into each cage and allowed to mate freely. Females were then blood fed and isolated for oviposition according to Axford et al. [26], and their progeny reared to adulthood and stored in absolute ethanol.

Ten progeny each from 30 isolated females per infection type were tested for the presence of *Wolbachia* using PCR to determine maternal transmission efficiency (see *DNA extraction and Wolbachia detection*). A set of control crosses was also completed for each infection type where both *Wolbachia*-infected females and uninfected males were reared under standard laboratory conditions. Ten progeny from 15 *Wolbachia*-infected females were tested for each of the *w*Mel, *w*AlbB and *w*MelPop infections. These crosses have expected maternal transmission rates of close to 100% [21–23]. All female parents from the treatments and controls were scored for their fecundity, with a sample also measured for wing length (see *wing length measurements*). Data from uninfected females reared under standard laboratory conditions from a concurrent experiment were included as a point of comparison.

### Wing length measurements

Linear measurements of wings were taken to give an indication of body size [74,75]. The right wing was removed from each adult and fixed on a slide under a 10 mm circular coverslip (Menzel-Gläser, Braunschweig, Germany) using Hoyer’s solution (dH_2_O: gum arabic: chloral hydrate: glycerin in the ratio 5: 3: 20: 2) [76]. Wings were observed under a dissecting microscope fitted with a camera and measured using NIS-Elements BR (Nikon Instruments, Japan). Wing length was determined by calculating the distance from the alular notch to the intersection of the radius 3 vein and outer margin, excluding the wing fringe scales [77]. Measurements in pixels were converted to millimetres by calibration with a graticule before the start of each set of measurements. Each measurement was repeated independently so that length represented the average of two measurements. Damaged or folded wings were excluded from the analysis.

### DNA extraction and *Wolbachia* detection

To test for the presence of *Wolbachia* in adult and immature mosquitoes, we carried out DNA extraction and *Wolbachia* detection according to methods described previously [24,26,78]. DNA from whole adults or larvae was extracted using 150 μL of 5% Chelex 100 resin (Bio-Rad Laboratories, Hercules, CA). The PCR assay was conducted using a LightCycler 480 system (Roche Applied Science, Indianapolis, IN); mosquitoes were considered positive for *Wolbachia* when the mRpS6 (*Aedes* universal) and aRpS6 (*Ae*. *aegypti*-specific) primer sets were successfully amplified in addition to the appropriate *Wolbachia*-specific primer set (*w*Mel, *w*AlbB or *w*MelPop). *Wolbachia*-free mosquitoes tested positive for mRpS6 and aRpS6 and negative for all *Wolbachia*-specific primer sets.

### Statistical analysis

All data were analysed using SPSS statistics version 21.0 for Windows (SPSS Inc, Chicago, IL). Survival data were investigated using Kaplan-Meier analysis; log-rank tests compared rates of mortality between lines and treatments. *Wolbachia* infection frequency was calculated as the proportion of individuals that tested positive for *Wolbachia*. For containers where both *Wolbachia*-infected and uninfected larvae were present, deviations from expected infection frequencies in larvae and adults were analysed using Chi-squared tests. Maternal transmission rates of *Wolbachia* were expressed as the proportion of infected offspring produced by infected mothers, for which 95% binomial confidence intervals were calculated. All other data were tested for normality using Shapiro-Wilk tests. Data that were not normally distributed were arcsine square-root transformed (proportional data) or square-root transformed and tested again. Normally distributed data were then analysed with one-way ANOVA and Tukey’s honest significant difference tests, while data that failed Shapiro-Wilk tests were analysed with non-parametric Kruskal-Wallis and Mann-Whitney U tests. Associations between wing length and development time were assessed with Pearson’s correlation if data were normally distributed or Spearman’s rank-order correlation where data could not be transformed for normality.

## Results

### Survival of isolated larvae under starvation conditions

Kaplan-Meier (KM) analysis revealed a significant effect of *Wolbachia* infection type (KM: χ^2^ = 123.273, df = 3, *P <* 0.0001) and water-replacement regime (KM: χ^2^ = 678.532, df = 1, *P* < 0.0001) on the survival of larvae when isolated under starvation conditions. Whether water was refreshed in each well or left unmanipulated had a dramatic effect on survival, with the former (mean ± SE = 20.682 ± 0.221 days) reducing the mean survival time of larvae by half compared with unmanipulated experimental wells (40.286 ± 0.573 days, S2 Fig). An increased survival in the latter experiment is likely due to the build-up of microorganisms which are an important resource for mosquito larvae [70,73,79].

**Fig 1:**
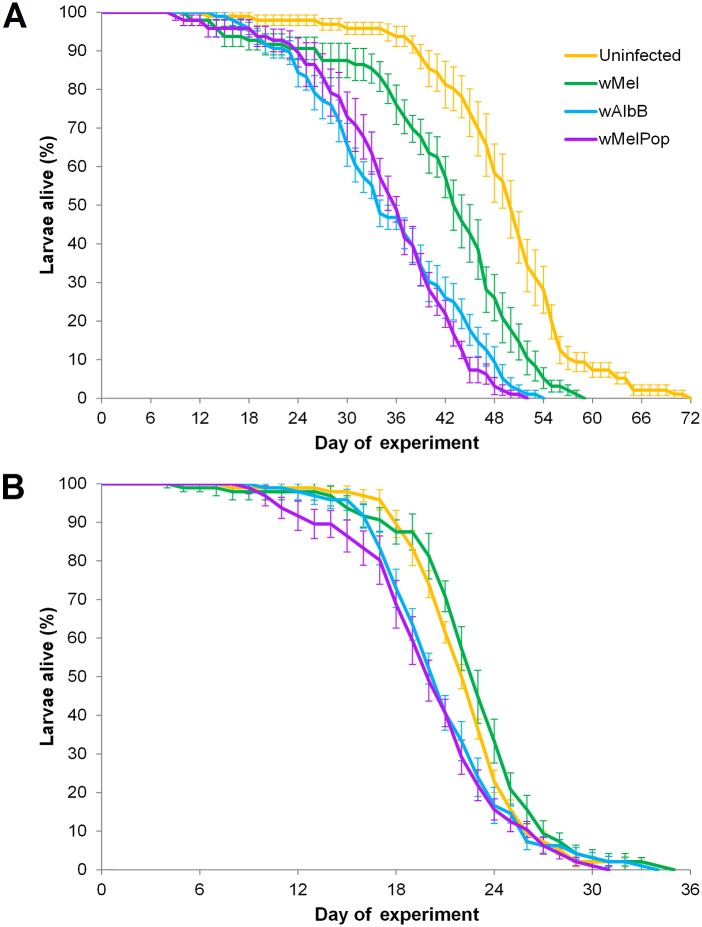
Survival of *Ae*. *aegypti* larvae when isolated under starvation conditions. (A) Survival of larvae when there was no manipulation of the wells. (B) Survival of larvae when the water in each well was replaced every four days. Error bars are standard errors. Note that (A) and (B) differ in their x-axis values.

When water was not replaced, all three *Wolbachia* infections reduced survival; the *w*Mel, *w*AlbB and *w*MelPop infections decreased mean survival 15.8, 28.8 and 28.7% compared with uninfected larvae (Fig 1A). All pairwise comparisons between the infection types were highly significant (KM: all χ^2^ > 24.087, df = 1, all *P <* 0.0001), with the exception that *w*MelPop and *w*AlbB did not differ significantly in their survival patterns under starvation conditions (χ^2^ = 0.717, df = 1, *P =* 0.397).

Although there was a significant effect of *Wolbachia* infection type in both experiments, survival differences between *Wolbachia*-infected and uninfected larvae were reduced markedly when water was replaced every four days (KM: χ^2^ = 17.939, df = 3, *P* = 0.0005) compared with wells that were unmanipulated (χ^2^ = 150.024, df = 3, *P* < 0.0001, Fig 1). When water was replaced, all pairwise comparisons between infection types were significant (KM: all χ^2^ > 4.262, df = 1, all *P* ≤ 0.039) except for between uninfected and *w*Mel (KM: χ^2^ = 1.707, df = 1, *P* = 0.191), and *w*AlbB and *w*MelPop (KM: χ^2^ = 0.630, df = 1, *P* = 0.427) (Fig 1B). No pupae or adults emerged in either experiment where larvae were isolated.

### Survival and development of larvae held in groups under starvation conditions

**Fig 2:**
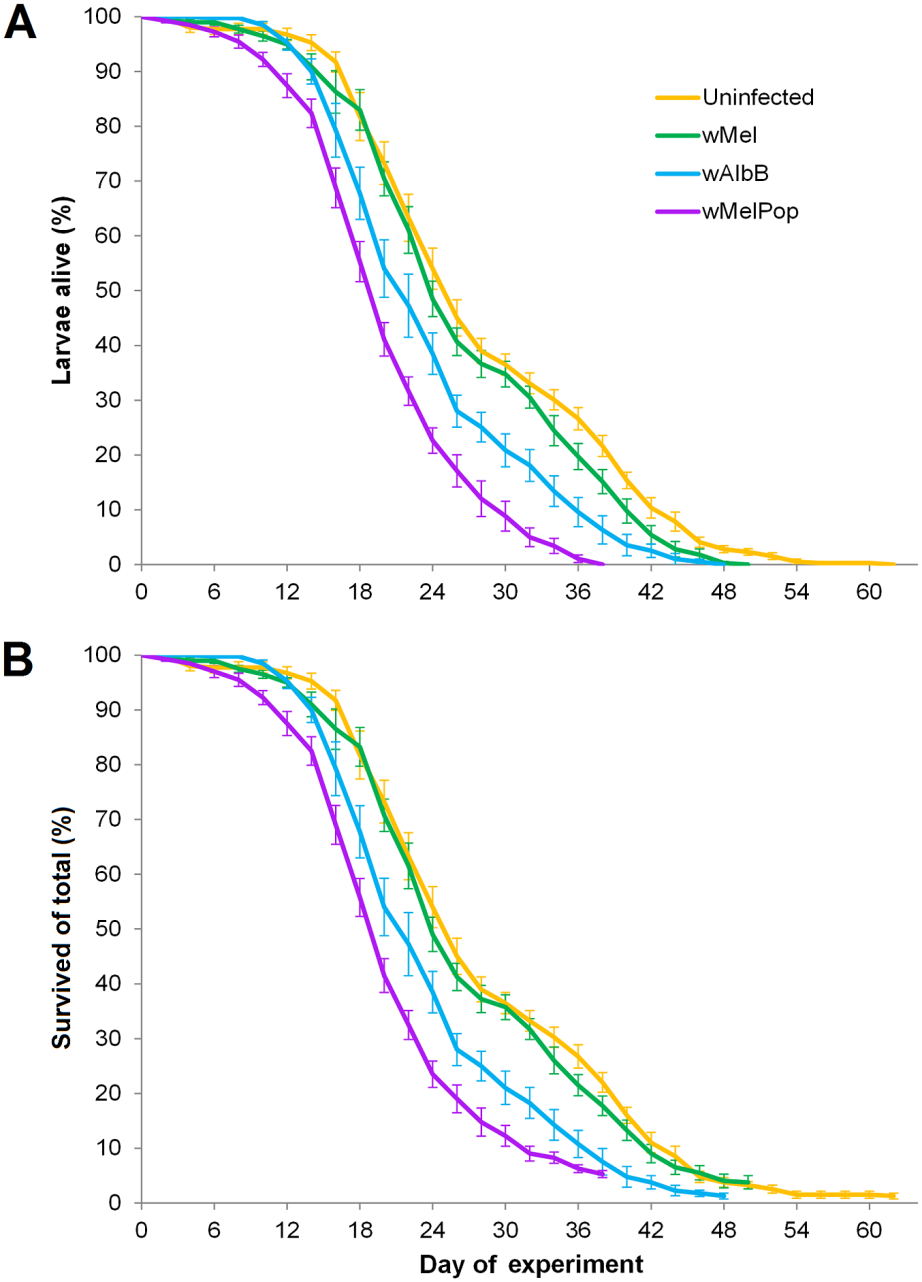
Survival of *Ae*. *aegypti* larvae under starvation conditions in groups of 50 per container. (A) Shows only larval mortality for each line and excludes those larvae that emerged as adults, while (B) is adjusted so that emerging adults are included in the survivors. Error bars are standard errors.

*Wolbachia* infection type also had a substantial effect on survival when larvae were held under starvation conditions in groups of 50 (KM: χ^2^ = 225.821, df = 3, *P* < 0.0001). Uninfected larvae had the greatest mean time of survival (mean ± SE = 28.289 ± 0.532 days), with the *w*Mel, *w*AlbB and *w*MelPop infections reducing survival times by 5.7, 15.7 and 29.5% respectively (Fig 2). All pairwise comparisons between lines were significant (KM: all χ^2^ > 7.411, df = 1, all *P* ≤ 0.006). Note that emerging adults were excluded from Kaplan-Meier analyses rather than censored because the rate and number of adults emerging differed between infection types.

Larvae from both *Wolbachia*-infected and uninfected lines readily consumed dead conspecifics throughout the experiment. We inferred scavenging based on observations that the number of dead larvae in each container fluctuated with mortality rather than increasing proportionally (S3 Fig). Distributions of necrophagy closely matched larval mortality, with the mean time for larval consumption occurring less than one day after the mean time of death for both *Wolbachia*-infected and uninfected lines (S4 Fig). Necrophagy likely contributed to increased survival time; larvae lived for longer in groups compared with larvae kept in isolation under otherwise similar conditions. While survival began to decline earlier in the group experiment, rates of mortality became considerably slower when the majority of larvae had died (S2 Fig).

Less than five percent of larvae reached pupation or adulthood during this experiment (Table 1). *Wolbachia* infection type had a significant effect on the total number of larvae that survived to both the pupal (one-way ANOVA: *F*_3, 28_ = 3.417, *P* = 0.031) and adult (*F*_3, 28_ = 5.647, *P* = 0.004) stages, and also affected the development times of those pupae (Kruskal-Wallis: χ^2^ = 31.499, df = 3, *P* < 0.0001) and adults (χ^2^ = 14.200, df = 3, *P* = 0.003). Despite uninfected larvae having greater survival times under starvation conditions (Fig 2), they developed more slowly and pupated less often than *Wolbachia*-infected larvae, with the *w*MelPop infection displaying the greatest proportion of larvae reaching adulthood and the most rapid development on average (Table 1, S5 Fig). This observation is likely due to an earlier availability and greater abundance of conspecific carcasses as a source of nutrition in containers with *w*MelPop-infected larvae.

**Table 1:** Pupation and adult emergence from *Ae*. *aegypti* larvae held under starvation conditions in groups of 50.

	Survival (%) ± SE		Development time (days) ± SE	
Infection type	Pupae*	Adults*	Pupae^†^	Adults^†^
Uninfected	2.25 ± 0.35 **a**	1.25 ± 0.26 **a**	43.33 ± 2.58 **a** (n = 9)	43.20 ± 3.93 **a** (n = 5)
*w*Mel	4.75 ± 0.80 **ab**	3.75 ± 0.61 **ab**	29.47 ± 3.19 **b** (n = 19)	29.47 ± 3.72 **ab** (n = 15)
*w*AlbB	3.00 ± 0.50 **ab**	1.25 ± 0.26 **a**	33.17 ± 0.87 **b** (n = 12)	33.20 ± 1.02 **b** (n = 5)
*w*MelPop	7.50 ± 0.53 **b**	5.25 ± 0.32 **b**	25.47 ± 1.45 **c** (n = 30)	26.86 ± 1.98 **c** (n = 21)

* Within a column, values with the same letter in bold are not significantly different from each other (*P* > 0.05, by Tukey’s honest significant difference test)

^†^ Within a column, values with the same letter in bold are not significantly different from each other (*P* > 0.05, by Mann-Whitney *U* tests on data pooled across replicates)

A second experiment was conducted where *Wolbachia*-infected and uninfected larvae were held together in the same container under starvation conditions. Control containers, where 48 larvae from each infection type were held separately, had a shorter starved survival period than in the previous experiment despite nearly identical methods, though the relative performance of each infection type was similar (S2 Fig). In each treatment container, the five longest-lived larvae were screened for their infection status to test for differential survival between infected and uninfected larvae when held together at different frequencies. The *w*AlbB and *w*MelPop infections were significantly underrepresented in the surviving larvae for all treatments, while for *w*Mel there were no significant deviations from any starting ratio (Table 2).

**Table 2:** *Wolbachia* infection frequencies in surviving *Ae*. *aegypti* larvae when held at different initial proportions under starvation conditions.

		Observed proportion *Wolbachia*-infected: uninfected								
Initial proportion *Wolbachia*-infected: uninfected		*w*Mel: uninfected			*w*AlbB: uninfected			*w*MelPop: uninfected		
Treatment*	Expected	Observed^†^	χ^2^^‡^	*P*^§^	Observed^†^	χ^2^^‡^	*P*^§^	Observed^†^	χ^2^^‡^	*P*^§^
36:12	30:10	30:10	0	1	23:17	6.53	**0.011**	12:28	43.20	**< 0.0001**
24:24	20:20	16:24	1.60	0.206	6:34	19.60	**< 0.0001**	6:34	19.60	**< 0.0001**
12:36	10:30	7:33	1.20	0.273	4:36	4.80	**0.029**	2:38	8.53	**0.004**
Total	60:60	53:67	1.63	0.201	33:87	24.30	**< 0.0001**	20:100	53.33	**< 0.0001**

* Cohorts of larvae were set up with initial ratios of 36:12, 24:24 and 12:36 (*Wolbachia*-infected: uninfected) and held under starvation conditions until five larvae per container were left alive.

^†^ Observed proportion of *Wolbachia*-infected: uninfected in the longest five surviving larvae of each container

^‡^ Chi-squared tests assessed deviations from expected ratios which were based on the initial proportion of *Wolbachia*-infected larvae in each container. Deviations from an expected 1:1 ratio were also tested when all treatments for each infection type were combined

^§^ *P*-values in bold denote significant deviations from expected ratios where all df = 1

Less than two percent of larvae from this experiment emerged as adults. Expected ratios of *Wolbachia*-infected and uninfected adults emerging were based on the initial proportion of larvae in each container. We found no significant deviations from expected proportions of adults for all treatments (Chi-squared test: all χ^2^ < 3.267, df = 1, all *P* > 0.071), except for the *w*MelPop infection which was significantly underrepresented when larvae were held in the ratio 36:12 (*w*MelPop: uninfected) (Chi-squared test: χ^2^ = 24.2, df = 1, *P* < 0.0001).

All adults that emerged from larvae held in groups were measured for wing length to test for effects on body size. Due to low numbers of adults, data were pooled across both experiments as they did not differ significantly (Student’s *t* test: *P* = 0.795). Wing length was not associated with development time for either males (Spearman’s rank-order correlation: *ρ* = 0.071, *P* = 0.455, n = 56) or females (*ρ* = -0.009, *P* = 0.924, n = 58). As expected, there was a significant effect of sex on wing length (one-way ANOVA: *F*_1,106_ = 285.910, *P* < 0.0001), where males (mean ± SE = 1.659 ± 0.009 mm) were considerably smaller than females (1.973 ± 0.015 mm). However, we found no effect of *Wolbachia* infection type (one-way ANOVA: *F*_3,106_ = 0.360, *P* = 0.782); wings of mosquitoes with any infection type were approximately the same size (Table 3).

**Table 3:** Wing lengths of *Ae*. *aegypti* adults emerging from groups of larvae held under starvation conditions.

	Wing length (mm) ± SE	
Infection type	Males	Females
Uninfected	1.657 ± 0.013 (n = 21)	1.973 ± 0.024 (n = 24)
*w*Mel	1.649 ± 0.019 (n = 11)	2.020 ± 0.024 (n = 8)
*w*AlbB	1.671 ± 0.021 (n = 7)	1.971 ± 0.042 (n = 10)
*w*MelPop	1.664 ± 0.020 (n = 17)	1.950 ± 0.027 (n = 16)

Data are pooled across experiments where infection types were held both separately and in mixed proportions. No values within a column differed significantly from each other by one-way ANOVA.

### Recovery from food deprivation

25.5% and 12.3% of larvae across all infection types survived after 15 and 25 days of exposure to starvation conditions respectively. *Wolbachia* infection type had a significant effect on the number of larvae surviving after both 15 (one-way ANOVA: *F*_3,56_ = 4.152, *P* = 0.010) and 25 days (*F*_3,26_ = 4.114, *P* = 0.016). The *w*MelPop infection had the lowest survival at both time points (S1 Fig), consistent with other experiments (Figs 1B and 2).

**Fig 3:**
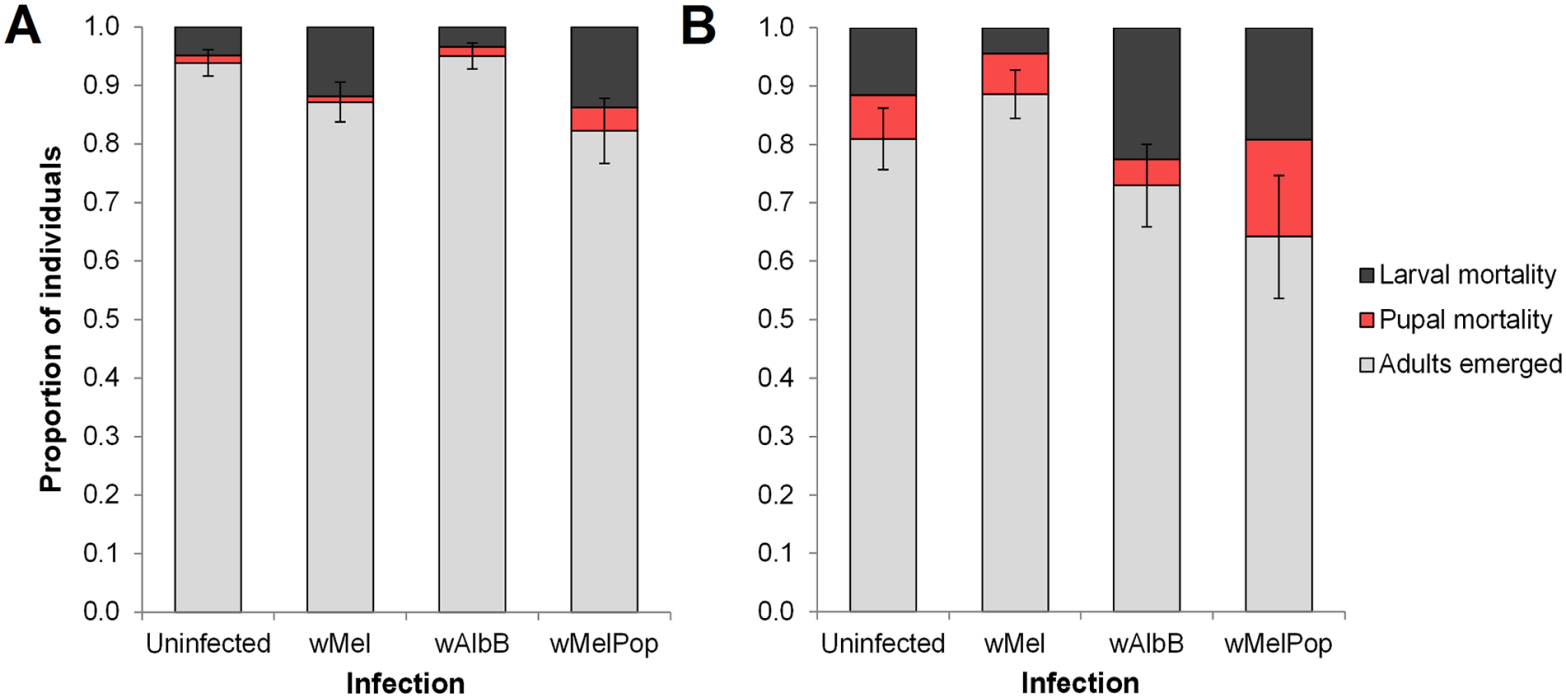
Proportion of *Ae*. *aegypti* larvae developing when fed *ad libitum* after extended food deprivation. Larvae were provided with TetraMin *ad libitum* after (A) 15 and (B) 25 days of food deprivation. Light grey bars denote the proportion of surviving larvae that reached adulthood, while black and red bars correspond to the proportion of larval and pupal mortality respectively. Error bars are standard errors for the proportion of larvae that survived to adulthood. Within treatments, no proportions differed significantly from each other (*P* > 0.05, by Tukey’s honest significant difference test).

Recovery from food deprivation was assessed by scoring the proportion of surviving larvae that pupated and reached adulthood upon resuming feeding. The majority of surviving larvae were able to recover, though larval and pupal mortality occurred across both treatments for all infection types (Fig 3). We found a significant effect of treatment (day of re-feeding) (one-way ANOVA: *F*_1,52_ = 5.576, *P* = 0.022), but not *Wolbachia* infection type (*F*_3,52_ = 1.461, *P* = 0.236), on the proportion of surviving larvae that reached adulthood. Surviving larvae that were deprived of food for 25 days were less likely to reach adulthood than larvae deprived for 15 days, with the percentage surviving of larvae that died after re-feeding averaging 10.4% and 22.9% respectively. This is, in part, due to an increase in pupal mortality at the later time point (2.1% for Day 15, 9.0% for Day 25, Student’s *t* test: *P* = 0.042, Fig 3). The proportion of surviving larvae that reached adulthood was less for *w*MelPop than for other infection types, though this difference was not significant (Fig 3). Larvae that reached pupation before re-feeding (33.3% of *w*MelPop-infected larvae and 3.3% of *w*Mel-infected larvae) were counted as survivors. However, these individuals were excluded from development time and wing length analyses (see below) as they pupated before food was provided again *ad libitum*, and were similar in size to adults emerging from larvae held in groups under starvation conditions (Table 3).

The number of days taken for larvae to reach pupation after re-feeding was significantly affected by infection type (one-way ANOVA: *F*_3, 488_ = 5.377, *P* = 0.001) but not treatment (day of re-feeding) (*F*_1, 488_ = 2.128, *P* = 0.145), though infection types within treatments did not differ significantly from each other (Table 4). Development times of both male and female adults were unaffected by infection type and treatment (one-way ANOVA: all *P* > 0.053). Female wing length was significantly affected by treatment (one-way ANOVA: *F*_1, 251_ = 6.696, *P* = 0.010) but not infection type (*F*_3, 251_ = 1.432, *P* = 0.234). Females re-fed after 25 days of food deprivation were smaller than those fed after 15 days for all infection types, though no pairwise comparisons were significant (Table 4). Conversely, male wing length was unaffected by both infection type (*F*_3, 194_ = 0.844, *P* = 0.471) and treatment (*F*_1, 194_ = 0.032, *P* = 0.859). We found no correlation between development time and wing length for both males and females for each treatment (Pearson correlation: all *P* > 0.175).

**Table 4:** Mean development time and wing length of *Ae*. *aegypti* when fed *ad libitum* after extended food deprivation.

	Development time (days after re-feeding) ± SE			Wing length (mm) ± SE	
Infection type	Pupae	Males	Females	Males	Females
**Re-fed on Day 15**					
Uninfected	4.302 ± 0.042 **ab** (n = 89)	6.075 ± 0.063 **a** (n = 47)	6.530 ± 0.105 **a** (n = 33)	2.192 ± 0.011 **a** (n = 43)	2.867 ± 0.022 **a** (n = 33)
*w*Mel	4.145 ± 0.022 **a** (n = 109)	5.877 ± 0.058 **a** (n = 54)	6.257 ± 0.074 **a** (n = 54)	2.185 ± 0.009 **a** (n = 54)	2.838 ± 0.016 **a** (n = 54)
*w*AlbB	4.379 ± 0.026 **ab** (n = 99)	5.953 ± 0.071 **a** (n = 38)	6.612 ± 0.084 **a** (n = 59)	2.192 ± 0.013 **a** (n = 35)	2.869 ± 0.018 **a** (n = 58)
*w*MelPop	4.309 ± 0.055 **ab** (n = 55)	6.129 ± 0.142 **a** (n = 21)	6.482 ± 0.149 **a** (n = 32)	2.161 ± 0.015 **a** (n = 21)	2.827 ± 0.022 **a** (n = 32)
**Re-fed on Day 25**					
Uninfected	4.478 ± 0.089 **b** (n = 42)	6.199 ± 0.256 **a** (n = 11)	6.883 ± 0.089 **a** (n = 29)	2.161 ± 0.020 **a** (n = 11)	2.831 ± 0.022 **a** (n = 29)
*w*Mel	4.264 ± 0.079 **ab** (n = 55)	6.140 ± 0.127 **a** (n = 26)	6.668 ± 0.086 **a** (n = 26)	2.194 ± 0.014 **a** (n = 25)	2.788 ± 0.014 **a** (n = 25)
*w*AlbB	4.404 ± 0.106 **ab** (n = 34)	6.354 ± 0.171 **a** (n = 8)	6.632 ± 0.139 **a** (n = 24)	2.196 ± 0.029 **a** (n = 7)	2.800 ± 0.027 **a** (n = 24)
*w*MelPop	4.250 ± 0.204 a**b** (n = 13)	5.876 ± 0.281 **a** (n = 6)	6.392 ± 0.311 **a** (n = 4)	2.169 ± 0.020 **a** (n = 6)	2.743 ± 0.084 **a** (n = 4)

Larvae were re-fed TetraMin *ad libitum* after either 15 (top) or 25 (bottom) days of food deprivation. Development time is defined as the number of days taken for larvae to reach pupation or adulthood after re-feeding. Within a column, values with the same letter in bold are not significantly different from each other (*P* > 0.05, by Tukey’s honest significant difference test).

### Cytoplasmic incompatibility, maternal transmission and fecundity when larvae are food-deprived then re-fed

Males deprived of food for 30 days as larvae and then re-fed were tested for their ability to induce cytoplasmic incompatibility when crossed to uninfected females. All food-deprived and re-fed *Wolbachia*-infected males exhibited complete cytoplasmic incompatibility, with no viable offspring produced across three gonotrophic cycles (Table 5). Control crosses using standard laboratory-reared adults were also completely sterile, with the exception that a low proportion of eggs hatched in the *w*MelPop control cross due to contamination with uninfected males (Table 5).

**Table 5:** Percentage of hatching eggs from crosses between *Wolbachia*-infected males and uninfected female *Ae*. *aegypti*.

	Gonotrophic cycle^†^		
Cross*	1	2	3
**Controls**^‡^			
Uninfected ♀ × *w*Mel ♂	0 (248.50 ± 25.67)	0 (247.38 ± 35.71)	0 (249.38 ± 41.28)
Uninfected ♀ × *w*AlbB ♂	0 (223.75 ± 24.56)	0 (188.625 ± 37.45)	0 (272.75 ± 18.25)
Uninfected ♀ × *w*MelPop ♂	0.30 (246.88 ± 22.96)	0.75 (234.63 ±14.30)	0.23 (221.75 ± 29.13)
**Treatments**^§^			
Uninfected ♀ × *w*Mel ♂	0 (298.00 ± 53.84)	0 (258.50 ± 21.18)	0 (206.88 ± 39.91)
Uninfected ♀ **×** *w*AlbB ♂	0 (272.75 ± 39.88)	0 (211.00 ± 20.71)	0 (194.38 ± 37.70)
Uninfected ♀ **×** *w*MelPop ♂	0 (174.88 ± 39.79)	0 (219.25 ± 34.71)	0 (184.38 ± 41.13)

* Eight cages with each containing seven males and seven females were tested per cross. All females were reared under standard laboratory conditions

^†^ Percentage hatch rates across three gonotrophic cycles are given, followed by the mean number of eggs laid per cross in parentheses, with standard errors

^‡^ *Wolbachia*-infected males were reared under standard laboratory conditions

^§^ *Wolbachia*-infected males were fed *ad libitum* as larvae for 72 hours, deprived of food for 30 days, then fed *ad libitum* until pupation

We also tested maternal transmission rates of *Wolbachia* when infected females were held under starvation conditions for 30 days as larvae and then re-fed. The *w*Mel, *w*AlbB and *w*MelPop infections were transmitted with perfect fidelity by both standard laboratory-reared females (All infection types: maternal transmission rate = 1, lower 95% binomial confidence interval = 0.976), and females that were food-deprived then re-fed (All infection types: maternal transmission rate = 1, lower 95% binomial confidence interval = 0.988).

Female parents were also measured for their fecundity and wing length. Both *Wolbachia* infection type (one-way ANOVA: *F*_3, 227_ = 33.011, *P* < 0.0001) and treatment (*F*_1, 227_ = 8.787, *P* = 0.003) had significant effects on fecundity. The food-deprivation treatment reduced the mean fecundity of *w*Mel, *w*AlbB and *w*MelPop-infected females by approximately 5–6 eggs relative to the controls, though no pairwise comparisons were significant (Table 6). All *Wolbachia*-infected females had considerably reduced fecundity compared with uninfected standard laboratory-reared females, regardless of the rearing treatment (Table 6). Female wing length was also significantly affected by both *Wolbachia* infection type (one-way ANOVA: *F*_3, 108_ = 6.935, *P* = 0.0003) and treatment (*F*_1, 108_ = 8.852, *P* = 0.004). For all infection types, females held under starvation conditions and then re-fed were smaller than standard laboratory-reared females, though only the *w*AlbB comparison was significant (Table 6).

**Table 6:** Average wing length and fecundity of isolated female *Ae*. *aegypti* tested for their maternal transmission fidelity.

Infection type	Feeding regime	Wing length (mm) ± SE*	Fecundity ± SE*
Uninfected	Control^†^	2.854 ± 0.018 **a** (n = 19)	68.21 ± 2.16 **a** (n = 39)
*w*Mel	Control^†^	2.757 ± 0.020 **abc** (n = 16)	50.68 ± 3.73 **b** (n = 22)
	Treatment^‡^	2.738 ± 0.027 **bc** (n = 16)	44.38 ± 2.34 **bc** (n = 42)
*w*AlbB	Control^†^	2.817 ± 0.019 **ab** (n = 16)	50.71 ± 2.46 **b** (n = 24)
	Treatment^‡^	2.688 ± 0.022 **c** (n = 16)	44.65 ± 1.81 **bc** (n = 43)
*w*MelPop	Control^†^	2.703 ± 0.021 **c** (n = 16)	38.86 ± 1.86 **cd** (n = 22)
	Treatment^‡^	2.665 ± 0.042 **c** (n = 16)	34.21 ± 1.36 **d** (n = 42)

* Within a column, values with the same letter in bold are not significantly different from each other (*P* > 0.05, by Tukey’s honest significant difference test)

^†^ *Wolbachia*-infected females were reared under standard laboratory conditions

^‡^ *Wolbachia*-infected females were fed *ad libitum* as larvae for 72 hours, deprived of food for 30 days, then fed *ad libitum* until pupation

## Discussion

We have demonstrated that *Wolbachia* infection reduces the tolerance of *Ae*. *aegypti* larvae to starvation conditions. Because *Ae*. *aegypti* larvae survive nutrient-poor conditions primarily by expending their own accumulated energy reserves [59,60], we suspect that *Wolbachia* reduce survival by increasing the rate at which these reserves are depleted. *Wolbachia* do not appear to affect the rate at which larvae accumulate reserves because development times are unaffected by infection when larvae are well-fed [26,37,38]. However, when food is limited, *Wolbachia* may increase the drain on host reserves due to various nutritional requirements [65–68]. Indeed, *Wolbachia* increase the metabolism of *Ae*. *aegypti* adults, at least for the *w*MelPop infection [42], though this remains to be tested in larvae.

All three infections negatively affected the survival patterns of nutrient-deprived larvae but differed in their severity; *w*MelPop was highly costly to survival across all experiments, *w*Mel either had a slightly deleterious or no significant effect relative to uninfected larvae, and *w*AlbB had an intermediate effect. These relative costs are consistent with their effects on mosquito adults and eggs; *w*MelPop drastically reduces adult lifespan and quiescent egg viability [21,37,38], *w*Mel has relatively minor costs or no detectable effect [22,24], and *w*AlbB has an intermediate cost to these traits [26]. Here, we demonstrate that infections with higher virulence in these life stages also have greater costs to the survival of larvae under starvation conditions. The differences between *Wolbachia* infections in terms of their deleterious effects are likely to be attributed to their density in mosquito tissues [80]. High bacterial densities and broad tissue tropisms in host cells are often implicated in increasing fitness costs imposed by *Wolbachia* infection, both in *Ae*. *aegypti* [22,26] and other insects [81–84].

We found that as the survival period of larvae increased, the deleterious effects of *Wolbachia* became clearer. In adults and eggs of *Ae*. *aegypti*, the fitness costs of *Wolbachia* are also enhanced with age; *w*MelPop has relatively little cost to the reproductive success of young females, but fecundity [38] and rates of successful probing [39,40] decline severely with subsequent gonotrophic cycles. Additionally, the *w*AlbB and *w*MelPop infections impose increased costs on the viability of quiescent eggs over time [26,37]. If these age effects also occur in larvae as suggested by our results, virulent *Wolbachia* infections could have difficulty invading populations where resources are scarce and thus development times are lengthened.

Adults emerging from starvation conditions were small in size, even in comparison with those produced through extreme crowding or nutrient limitation (e.g. [75,85,86]). Adult sizes were at the lowest end of natural variation found in Australian field populations of *Ae*. *aegypti*, from where these mosquitoes were sourced [87,88]. Adult body size reflects the feeding history of larvae after reaching a critical weight [54]; therefore adults emerging from starvation conditions likely obtained only the minimum nutritional reserves required for pupation. In contrast, larvae that were deprived of food for extended durations and then fed *ad libitum* emerged nearly as large as mosquitoes fed *ad libitum* throughout development, suggesting that they were able to attain a close approximation of their maximum weight despite the long interruption to feeding [52,53,55,58].

We found that *Ae*. *aegypti* larvae, regardless of *Wolbachia* infection type, recover well from long periods of nutrient deprivation. While the ability of larvae to resume their development has been reported previously [54,56,59], we show that larvae exhibit low mortality, pupate rapidly and emerge at a large size when fed again after being deprived of food for as long as three weeks. In addition, infected males deprived of food as larvae for one month exhibited complete cytoplasmic incompatibility and females transmitted *Wolbachia* to their offspring with perfect fidelity despite a greatly extended development time. Maternal transmission rates of *Wolbachia* also remain high when eggs are held in a quiescent state for several weeks [41]. In insects, the maternal transmission efficiency of *Wolbachia* [35,89–91] and the strength of cytoplasmic incompatibility [36,92–95] are known to be affected by bacterial density. Because environmental factors such as temperature [96–98] and nutrition [68,90,99,100] modulate *Wolbachia* density, extreme stress in the field could lead to changes in host effects derived from *Wolbachia*. However, the *w*Mel infection of *Ae*. *aegypti* established in Australian field populations has so far remained stable in terms of its reproductive effects, fitness costs and dengue blockage [24,101].

We acknowledge some limitations of our laboratory study that should be addressed in future experiments. We were somewhat limited in our ability to discern any effects of *Wolbachia* on larval development time and survival to adulthood when held under starvation conditions, due to low pupation rates. Future experiments testing these traits specifically should use larger cohorts with greater replication. Furthermore, we demonstrated the fitness costs of *Wolbachia* under rather arbitrary and specific scenarios. Nutrient input in the field is dynamic [102], but in this study larvae were fed for a single time period before either being deprived of food completely or re-fed at a later point. Breeding containers in the field are often populated by multiple cohorts [45,103,104], and Suh and Dobson [43] recently reported differential survival of *Wolbachia*-infected and uninfected 1^st^ instar *Ae*. *aegypti* larvae in the presence of later instars. Because predatory behaviour is more likely to occur under nutrient-poor conditions [64], future experiments on survival under starvation conditions should also test interactions between larvae of mixed age classes. Our experiments also were conducted over multiple generations, and while all infection types were outcrossed to an uninfected colony, the number of generations spent in the laboratory varied between experiments. Laboratory adaptation can have substantial effects on fitness [6,105], which could explain why larvae in some experiments had reduced survival under similar conditions (see S2 Fig)

Nevertheless, our study demonstrates consistent deleterious effects of *Wolbachia* on the survival of *Ae*. *aegypti* larvae under starvation conditions. To predict the impact on the invasion dynamics of *Wolbachia* in highly resource-limited habitats, we estimate changes to the unstable equilibrium frequency, denoted $\hat{p}$, when this cost to larval viability is considered. For *Wolbachia* to reach fixation in a population its frequency must reach or exceed $\hat{p}$; larger $\hat{p}$ values thus decrease the likelihood and speed of invasion, and will additionally reduce the potential for spatial spread once established in a population [31,106,107].

Based on the mean survival time of larvae under starvation conditions (averaged across all experiments where larvae were held in groups), we estimate the relative fitness of the *w*Mel, *w*AlbB and *w*MelPop infections to be 92.3, 81.3 and 68.5% that of uninfected respectively. We detected no significant costs for other traits, thus only the cost to survival patterns under starvation conditions is considered. Following equation 17b of Turelli [108], this produces a $\hat{p}$ of 0.08, 0.19 and 0.32 for *w*Mel, *w*AlbB and *w*MelPop respectively in the absence of any other fitness costs, assuming complete cytoplasmic incompatibility and no maternal transmission leakage as indicated by our results. Previous laboratory studies have estimated the fitness costs of the *w*Mel, *w*AlbB and *w*MelPop infections to be approximately ~24% [22], ~15% [23,26] and ~43% [37,108] respectively. Using these estimates, $\hat{p}$ increases to 0.30, 0.31 and 0.61 for *w*Mel, *w*AlbB and *w*MelPop respectively when both the costs to larval viability under starvation conditions and deleterious effects on other life stages are considered.

In a more extreme scenario, where larvae are deprived of food for 25 days before being provided access to food *ad libitum*, the invasive potential of *Wolbachia* decreases further. Assuming *Wolbachia*-infected larvae are equally as capable of recovering from food deprivation as suggested by our results, the relative fitness of the *w*Mel, *w*AlbB and *w*MelPop infections decrease to 90.6, 73.9 and 42.5% that of uninfected respectively. This corresponds to increases of $\hat{p}$ to 0.31, 0.37 and 0.75 when taking into account other fitness costs. The deleterious effects demonstrated here could in part explain why *w*MelPop was able to establish in semi-field cages [22,41] but has had great difficulty invading wild mosquito populations, both in Australia and Vietnam [50]. In semi-field cages, any costs of *Wolbachia* infection to larval viability under nutrient stress were likely to be masked by the fact that larvae were relatively well-fed. On the other hand, survival of larvae under starvation conditions was likely to be a critical fitness component in the field releases. The deleterious effects of *Wolbachia* demonstrated here will, therefore, have an impact on the potential for these infections to invade natural mosquito populations where competition for resources is the major limiting factor of population size, particularly for *w*MelPop.

## Supporting Information

S1 Appendix Horizontal transfer of *Wolbachia* through necrophagy of conspecific larvae. (DOCX)

S1 Fig Survival of *Ae*. *aegypti* larvae under starvation conditions during the recovery from food deprivation experiment. Points A and B denote when larvae were re-fed TetraMin for the experiment. Survival curves are based on 12–16 replicates for each line until Day 15 and 6–8 replicates after Day 15. Error bars are standard errors.
(TIF)

S2 Fig Comparison of larval survival under starvation conditions between experiments. Larvae of *Ae*. *aegypti* were held under starvation conditions in isolation when water was replaced every four days (solid red line) or when water was left unmanipulated (dashed red line). Experiments where larvae were held in groups (grey lines) were conducted under similar conditions (water was replaced), but the mixed cohort (dashed grey line) and recovery (dotted grey line) experiments were conducted at a later time on different generations. Data are averaged across all four infection types. Error bars are standard errors.
(TIF)

S3 Fig Larval mortality and dead larvae observed when *Ae*. *aegypti* larvae were held in groups under starvation conditions. Rates of larval mortality are shown by solid lines while the numbers of dead larvae observed are shown by dotted lines. The dotted line being below the solid line suggests that mortality is occurring at a slower rate than the consumption of larvae.
(TIF)

S4 Fig Larval mortality of *Ae*. *aegypti* and the number of larvae consumed when held in groups under starvation conditions. Rates of larval mortality are shown by solid lines while the numbers of dead larvae inferred to be consumed are shown by dashed lines. The delay between distributions of larval mortality and consumption provide an estimate of the rate of necrophagy in group containers. Mean delays between mortality and consumption are as follows: Uninfected, 0.60 days; *w*Mel, 0.32 days; *w*AlbB, 0.44 days; *w*MelPop, 0.81 days.
(TIF)

S5 Fig Pupae and adults of *Ae*. *aegypti* observed when larvae were held under starvation conditions in groups of 50. Number of (A) pupae and (B) adults emerging in total from eight containers of 50 larvae for each infection type.
(TIF)
