# Supplementary material for: Costs of Three Wolbachia Infections on the Survival of Aedes aegypti Larvae under Starvation Conditions
Source: PLoS Negl Trop Dis. 2016 Jan 8;10(1):e0004320. doi: 10.1371/journal.pntd.0004320 (PMC4706305; doi:10.1371/journal.pntd.0004320)
Supplement: S1 Appendix — (DOCX) [file pntd.0004320.s001.docx]

**S1 Appendix. Horizontal transfer of *Wolbachia* through necrophagy of conspecific larvae**

*Wolbachia* infections can be transferred horizontally to naturally uninfected arthropods through the consumption of infected individuals, both intra- and inter-specifically [[1-4](#_ENREF_1)]. While there is no evidence that *Wolbachia* from *Ae. aegypti* can be transferred to distantly related predators through consumption [[5](#_ENREF_5)], intra-specific transfer facilitated through the necrophagy of *Ae. aegypti* larvae is yet to be tested. *Ae. aegypti* larvae will readily consume dead conspecifics in the absence of higher quality nutrition [[6](#_ENREF_6),[7](#_ENREF_7)] and may even cannibalise earlier instars [[8](#_ENREF_8)], therefore the potential exists for intra-specific horizontal transfer to occur during field releases of *Wolbachia*-infected mosquitoes.

We tested whether *Wolbachia* infections could be transferred to uninfected larvae through direct consumption in two experiments. In the first, 24 hour old uninfected larvae were transferred to containers of RO water. Every two days, *w*MelPop-infected larvae of approximately the same size as uninfected larvae were fatally wounded and immediately added to the containers of uninfected larvae. Uninfected larvae were fed *w*MelPop-infected larvae *ad libitum* for 5-6 days and then stored in absolute ethanol. In the second experiment this method was repeated, but uninfected larvae were reared to adulthood and aged for one week before being stored in ethanol for *Wolbachia* detection. 20 4^th^ instar larvae and 20 adults (whole bodies) reared exclusively on a diet of *w*MelPop-infected larvae were screened for their infection status (see *DNA extraction and Wolbachia detection*). To test if *Wolbachia* could be detected in the carcasses being consumed, we also screened dead *w*MelPop-infected larvae held in containers of water for 0, 36 and 72 hours (n = 16 per time point).

We found no evidence that *Wolbachia* infections could be transferred through the consumption of infected conspecifics. No traces of *Wolbachia* in uninfected larvae were detected when they were fed exclusively *w*MelPop-infected larvae. The ability to detect *Wolbachia* in carcasses declined rapidly; 100, 63 and 38% of *w*MelPop-infected larvae tested positive for *Wolbachia* when killed and left in water for 0, 36 and 72 hours respectively. While this could in part explain the inability to detect horizontal transmission, scavenging of carcasses in other experiments occurred rapidly (S5 Fig.). These results suggest that horizontal transfer is unlikely to occur in the field and thus will not affect invasion dynamics. The results of experiments in which infected and uninfected larvae are present in the same container are also are unlikely to be confounded by any false positives for *Wolbachia* in screened larvae or adults.

**References**

1. Heath BD, Butcher RD, Whitfield WG, Hubbard SF (1999) Horizontal transfer of *Wolbachia* between phylogenetically distant insect species by a naturally occurring mechanism. Curr Biol 9: 313-316.

2. Wu K, Hoy MA (2012) Extended starvation reduced and eliminated *Wolbachia*, but not *Cardinium*, from *Metaseiulus occidentalis* females (Acari: Phytoseiidae): a need to reassess *Wolbachia*'s status in this predatory mite? J Invertebr Pathol 109: 20-26.

3. Le Clec'h W, Chevalier FD, Genty L, Bertaux J, Bouchon D, et al. (2013) Cannibalism and predation as paths for horizontal passage of *Wolbachia* between terrestrial isopods. PLoS One 8: e60232.

4. Brown AN, Lloyd VK (2015) Evidence for horizontal transfer of *Wolbachia* by a *Drosophila* mite. Exp Appl Acarol 66: 301-311.

5. Hurst TP, Pittman G, O'Neill SL, Ryan PA, Le Nguyen H, et al. (2012) Impacts of *Wolbachia* infection on predator prey relationships: evaluating survival and horizontal transfer between *w*MelPop infected *Aedes aegypti* and its predators. J Med Ent 49: 624-630.

6. Daugherty MP, Alto BW, Juliano SA (2000) Invertebrate carcasses as a resource for competing *Aedes albopictus* and *Aedes aegypti* (Diptera: Culicidae). J Med Ent 37: 364-372.

7. Bara J, Clark T, Remold S (2014) Utilization of larval and pupal detritus by *Aedes aegypti* and *Aedes albopictus*. J Vector Ecol 39: 44-47.

8. Edgerly J, Willey M, Livdahl T (1999) Intraguild predation among larval treehole mosquitoes, *Aedes albopictus*, *Ae. aegypti*, and *Ae. triseriatus* (Diptera: Culicidae), in laboratory microcosms. J Med Ent 36: 394-399.
