# Supplementary figures and images for: Costs of Three Wolbachia Infections on the Survival of Aedes aegypti Larvae under Starvation Conditions
Source: PLoS Negl Trop Dis. 2016 Jan 8;10(1):e0004320. doi: 10.1371/journal.pntd.0004320 (PMC4706305; doi:10.1371/journal.pntd.0004320)

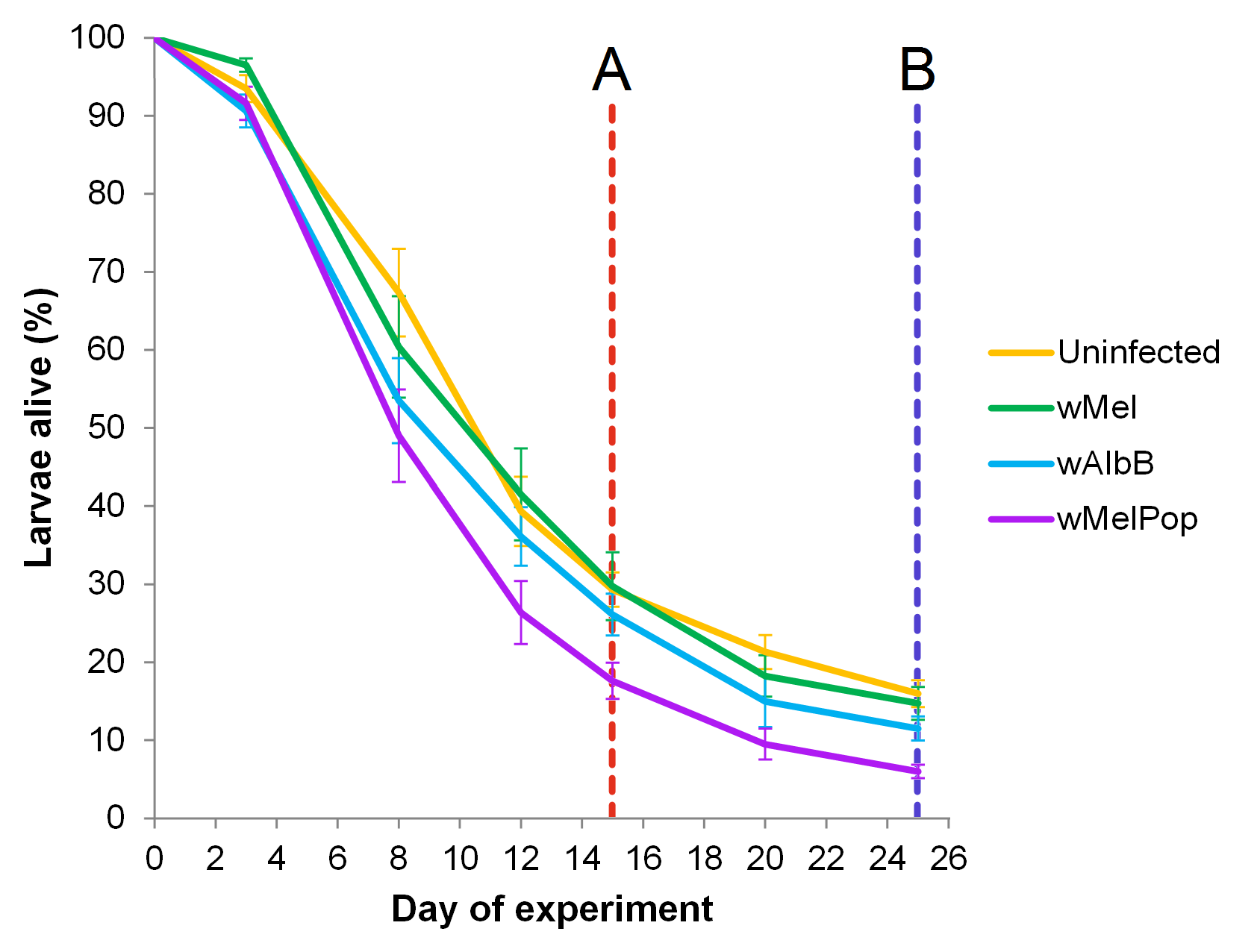

Supplement: S1 Fig — Points A and B denote when larvae were re-fed TetraMin for the experiment. Survival curves are based on 12–16 replicates for each line until Day 15 and 6–8 replicates after Day 15. Error bars are standard errors. (TIF) [file pntd.0004320.s002.tif]

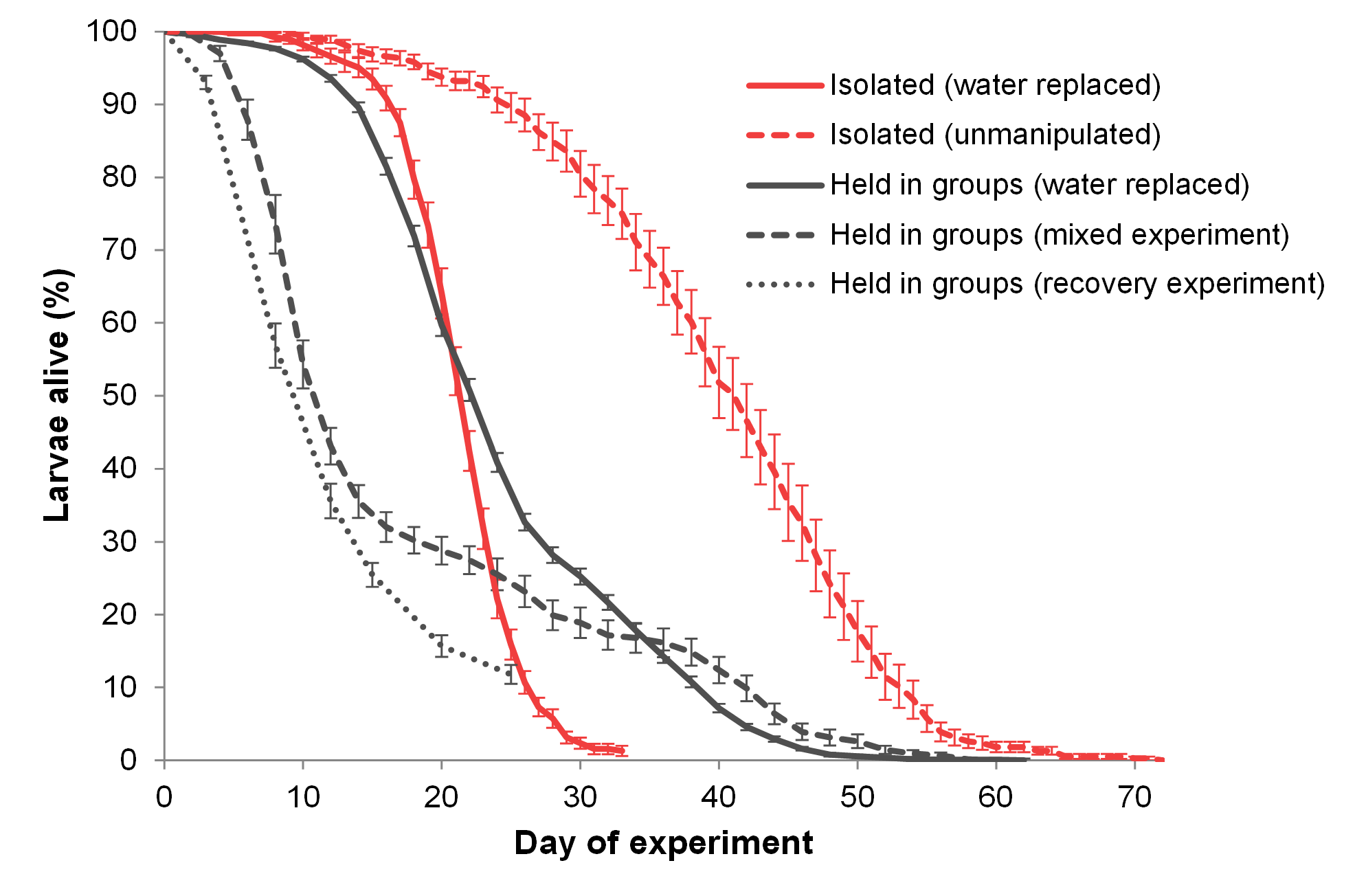

Supplement: S2 Fig — Larvae of Ae. aegypti were held under starvation conditions in isolation when water was replaced every four days (solid red line) or when water was left unmanipulated (dashed red line). Experiments where larvae were held in groups (grey lines) were conducted under similar conditions (water was replaced), but the mixed cohort (dashed grey line) and recovery (dotted grey line) experiments were conducted at a later time on different generations. Data are averaged across all four infection types. Error bars are standard errors. (TIF) [file pntd.0004320.s003.tif]

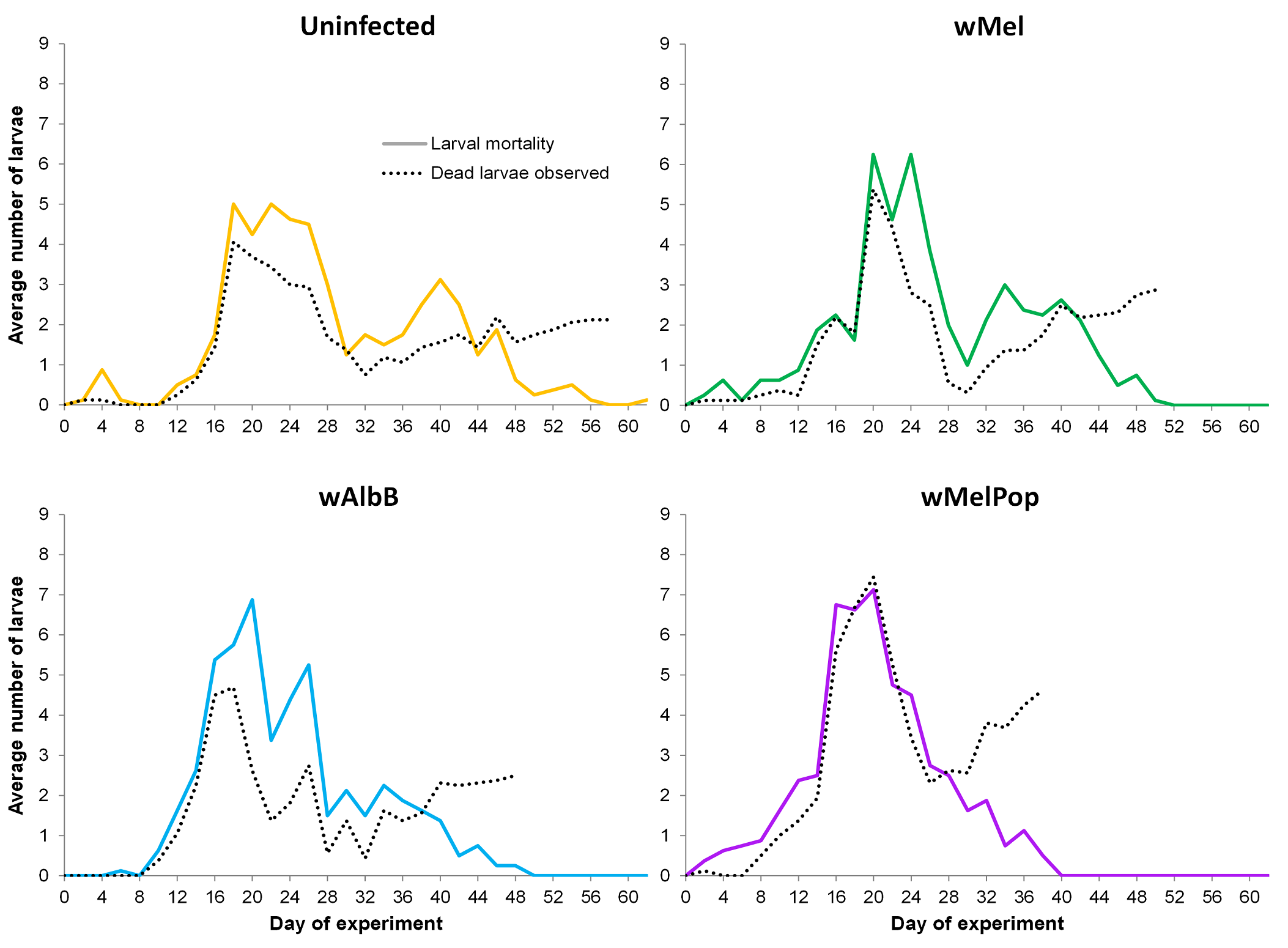

Supplement: S3 Fig — Rates of larval mortality are shown by solid lines while the numbers of dead larvae observed are shown by dotted lines. The dotted line being below the solid line suggests that mortality is occurring at a slower rate than the consumption of larvae. (TIF) [file pntd.0004320.s004.tif]

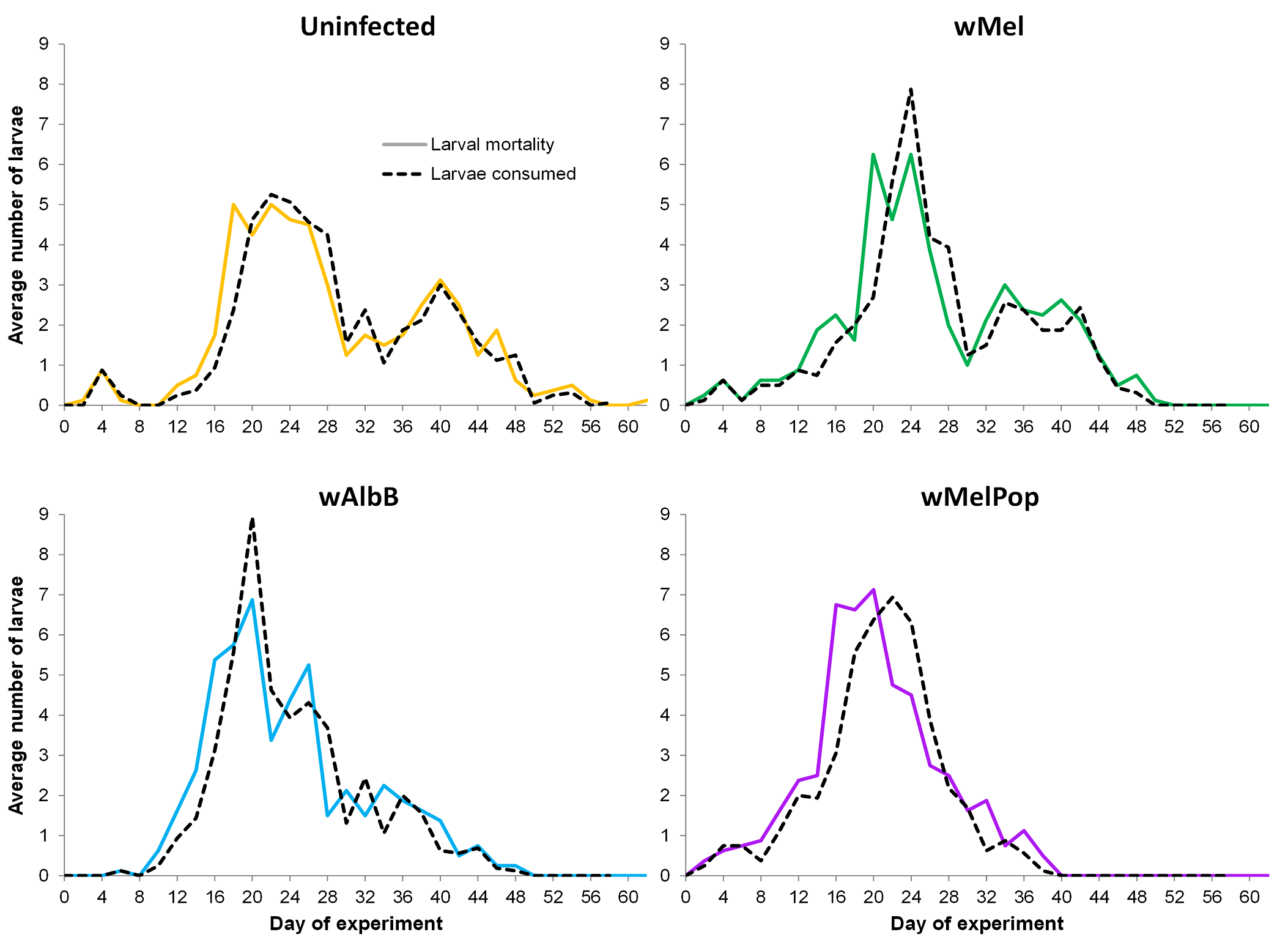

Supplement: S4 Fig — Rates of larval mortality are shown by solid lines while the numbers of dead larvae inferred to be consumed are shown by dashed lines. The delay between distributions of larval mortality and consumption provide an estimate of the rate of necrophagy in group containers. Mean delays between mortality and consumption are as follows: Uninfected, 0.60 days; wMel, 0.32 days; wAlbB, 0.44 days; wMelPop, 0.81 days. (TIF) [file pntd.0004320.s005.tif]

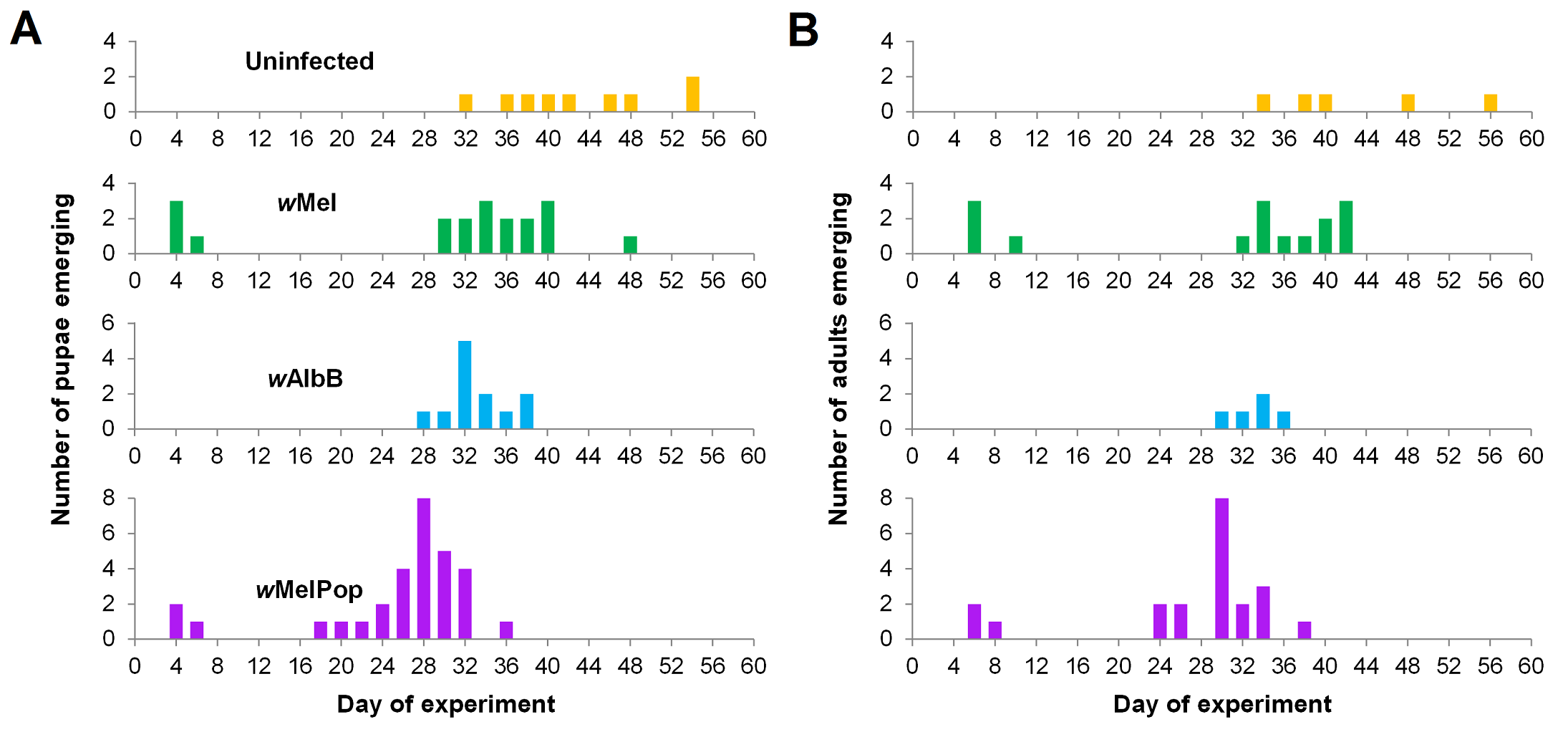

Supplement: S5 Fig — Number of (A) pupae and (B) adults emerging in total from eight containers of 50 larvae for each infection type. (TIF) [file pntd.0004320.s006.tif]
